# Nutrition, Energy Expenditure, Dysphagia, and Self-Efficacy in Stroke Rehabilitation: A Review of the Literature

**DOI:** 10.3390/brainsci8120218

**Published:** 2018-12-07

**Authors:** Adam C. Lieber, Estee Hong, David Putrino, Dominic A. Nistal, Jonathan S. Pan, Christopher P. Kellner

**Affiliations:** 1 Department of Neurosurgery, Icahn School of Medicine at Mount Sinai, New York, NY 10029, USA; adam.lieber@icahn.mssm.edu (A.C.L.); dominic.nistal@icahn.mssm.edu (D.A.N.); jonathan.pan@icahn.mssm.edu (J.S.P.); 2 Department of Rehabilitation Medicine, Icahn School of Medicine at Mount Sinai, New York, NY 10029, USA; eh469@cornell.edu (E.H.); dputrino83@gmail.com (D.P.)

**Keywords:** neuroprotection, nutrition, stroke, dysphagia, lifestyle, primary prevention, diet and nutrition, ischemic stroke, intracranial hemorrhage

## Abstract

While significant research has been performed regarding the use of thrombolytic agents and thrombectomy in the setting of acute stroke, other factors, such as nutritional status of stroke patients, is a less explored topic. The topic of nutrition is critical to the discussion of stroke, as up to half of stroke survivors may be considered malnourished at discharge. Dysphagia, old age, restricted upper limb movement, visuospatial impairment, and depression are all important risk factors for malnutrition in this cohort. The purpose of this review is to analyze current literature discussing neuroprotective diets, nutritional, vitamin, and mineral supplementation, dysphagia, and post-stroke coaching in stroke patients.

## 1. Introduction

Stroke is the second leading cause of death worldwide, and the prevalence of stroke and related deaths is increasing [1]. Many studies highlight the neuroprotective benefits of a diet high in fruits and vegetables or in minerals such as potassium [2,3]. Nutritional counseling and improvement may also play a critical role for stroke patients, as rehabilitation outcomes, mortality, and length of hospitalization are closely tied to patient nutritional status [4,5,6]. However, the majority of stroke patients do not meet estimated average requirements (EAR) and are susceptible to nutritional deterioration and significant body tissue loss [5,7,8].

Previous studies have assessed stroke survivor nutritional status at discharge and have reported frequencies of malnutrition ranging from 6.1% to 49% [9,10,11,12,13,14]. Many risk factors contribute to malnutrition among stroke patients, including dysphagia, restricted upper limb movement, visuospatial impairment, and depression [15,16]. Additionally, older cohorts are more prone to nutritional deficiency due to changes in physiological function efficiency, chronic illness, and social isolation [17,18].

Approximately 80% of neurological recovery occurs within 30 days after an acute ischemia event [19]. Therefore, early detection of malnutrition onset is critical to improve functional independence measure (FIM) efficiency and patient outcomes [20,21,22]. Patient screening using nutritional screening scores such as, Geriatric Nutritional Risk Index (GNRI), Malnutrition Universal Screening Tool (MUST), anthropometric indices such as body mass index (BMI) of 19 kg/m^2^ or lower, and prealbumin levels might prove to be efficient screening methods to detect malnutrition [23]. Upon detection of impending or current patient malnourishment, various nutritional interventions may help improve patient nutritional status.

During the rehabilitation phase, patients experiencing impaired balance control or participating in mobility-related activities require larger energy requirements to execute motor activities [24,25]. An increase in stroke patient resting energy expenditure (REE) is also observed, particularly in patients with subarachnoid hemorrhage (SAH) [26]. On average, hospitalized, acute stroke patients are reported to consume 80% to 91% of energy requirements [27]. Increased energy requirements and elevated REE in combination with failure to meet daily energy requirements will likely result in negative energy balance.

Prior preclinical rodent models positively associate intermittent fasting (IF) or reduction in dietary energy intake to prevent neurological damage from ischemic stroke and improve recovery [28,29]. In calorie-restricted aged rats, weight loss was associated with improved behavioral recovery and spatial memory [30].

Opposing evidence suggests that negative energy balance and weight loss are associated with poor functional recovery in patients with cerebrovascular disorders [23]. Following clinical evidence, calorie supplementation may benefit stroke patients by preventing negative energy balance due to higher energy expenditure and REE.

Along with energy supplementation, nutritional intervention can further supplement stroke rehabilitation and improve recovery outcomes. Numerous clinical studies support the positive impact of nutritional improvement. Nutritional improvement can include enhanced nutrient intake, various types of dietary supplementation, and mineral and vitamin supplementation. For malnourished patients, intensive nutritional supplementation was associated with greater motor recovery than standard nutritional supplementation [31]. In comparison to the control group, the supplemented group showed a significant increase in energy intake and protein intake with a trend to lower mortality [32,33]. To reduce damage from brain infarctions and stabilize neuronal function, particular emphasis on protein intake is highly essential to increase amino acid availability and support protein synthesis [19]. Vitamin and mineral supplementation was found to prevent and reverse neurological damage.

The purpose of this review is to analyze current literature discussing patient diet after stroke onset. The effect of dietary intake both within and outside the hospital setting will be evaluated during the immediate weeks post-stroke and over the long-term recovery process. Although there is a body of literature that investigates the role of nutritional supplementation as a method of primary prevention for stroke and cardiovascular disease (VITATOPS, HOPE-2, VISP, CSPPT, etc.), only current literature relevant to acute and subacute stroke recovery will be discussed [34,35,36,37,38,39,40]. 

## 2. Does the Energy Expenditure of Stroke Patients Differ from that of the General Population?

The question of whether a stroke changes the resting energy expenditure (REE) of a patient is controversial and studies offer conflicting evidence. In a study of 91 stroke patients, Finestone et al. calculated REE at 7, 11, 14, 21, and 90 days post-stroke by indirect calorimetry. It was found that stroke patients did not have a different REE than controls, and that there were no associations between stroke characteristics and REE [41]. However, one drawback of this study was that while the REE was measured for the stroke patients at many time points, the control patients only had one measurement performed. In a cross-sectional study of 95 patients in a subacute rehabilitation unit, Kawakami et al. also found that there were no significant differences in REE in subacute stroke patients versus controls (stroke patients averaged 1271 ± 284 kcal/day while controls averaged 1128 ± 231 kcal/day, *p* = 0.18). However, it was found that left hemispheric strokes were negatively correlated with REE, which Kawakami et al. hypothesized may be a result of dominant hand dysfunction and the association of left hemispheric strokes and depression [42].

In contrast, in a pilot study of 67 patients, Leone and Pencharz found that mean REE in 10 stroke patients on enteral nutrition were significantly lower than 57 controls (*p* < 0.02). However, the small sample size of the stroke cohort is a limitation of the study [43]. Finally, a study by Nagano et al. measured REE by indirect calorimetry in 12 patients who underwent endovascular coiling of a ruptured aneurysm associated with subarachnoid hemorrhage (SAH) and 30 patients with acute cerebral infarction (ACI). The study found that the REE demands of the SAH group were significantly elevated by 12% compared to the predicted value given by the Harris–Benedict equation (*p* < 0.05), suggesting that either the disease process or the surgical intervention may have caused this increase [26]. Understanding the role of stroke in resting energy expenditure is vital to ensuring that the post-stroke patient is receiving adequate nutrition.

In addition to the question of the resting energy expenditure of stroke patients, researchers have sought to quantify the energy expenditure of stroke patients when performing certain motor tasks. Houdijk et al. found that 10 stroke patients had an average of 125% more energy expenditure than 12 controls in various standing balance tasks (*p* < 0.05), likely due to the impact stroke has on vestibular function and the focus required to overcome these deficits [24]. Additionally, Serra et al. found that stroke patients consumed higher oxygen levels during physical activity such as stepping in place, walking on ground, and walking on the treadmill relative to resting oxygen consumption compared to healthy controls (*p* < 0.05) [25].

Stroke survivors often face muscle atrophy from physical disability, both temporary or permanent, as well as continual loss of muscle mass due to aging. Muscle atrophy weakens muscle strength and exercise capacity, greatly impacting daily activities, post-stroke recovery, and metabolic requirements [44,45]. Studies have reported conflicting evidence on the effect of muscle atrophy on REE. Serra, Hafer-Macko, & Ryan evaluated resting metabolic rate (RMR) and body composition of stroke survivors with chronic hemiparesis (*n* = 39, mean age 61 ± 1 years) with indirect calorimetry and dual-energy X-ray absorptiometry (DXA), respectively. Subject RMR was compared to predicted RMR values obtained with the Mifflin–St Jeor equation. RMR values of stroke survivors with chronic hemiparesis (*n* = 39, mean age 61 ± 1 years) were 14% lower than predicted RMR values. While subject age or latency from stroke were not associated with RMR, total body and total leg lean tissue mass predicted RMR. Therefore, muscle atrophy may be associated with reduced resting metabolic rate (RMR) [46]. Alternatively, Tacke et al. measured fat-free mass (FMM) by DXA and REE by indirect calorimetry in 166 patients with chronic heart failure, 34 of which experienced muscle atrophy, and 27 healthy controls. Compared to patients with muscle atrophy (mean age 67.4 ± 10.2 years), controls and patients without muscle atrophy had significantly lower REE (1748 ± 359 kcal/d, 1579 ± 289 and 1532 ± 265, *p* = 0.018 and *p* = 0.001, respectively) [47].

## 3. Are Stroke Patients Ingesting enough Calories in the Hospital and Rehabilitation Settings?

Strong evidence exists that a large proportion of stroke patients do not meet their estimated energy requirement both in the hospital and after discharge. In a consecutive cohort study of 36 patients admitted to a South London hospital with acute stroke in 2001, the mean energy intake during the hospital stay was 60% of their estimated average requirement, improving only to 81% at 6 months [8]. In another consecutive cohort study of 100 acute stroke patients in a metropolitan hospital, a greater percentage of patients had energy intakes of <50% of their estimated average requirement (33%) than patients who met their energy requirement (10%) within 2 weeks of admission [5]. In a third study evaluating regular, dysphagia, and enteral tube diets, the average energy intake of the entire group at four different points in the hospital stay (day 7, 11, 14, and 21) ranged from 80.3–90.9% of the energy requirements, with enteral tube diets proving most sufficient [27]. When considering these data, it is clear that calorie requirements are not met by stroke patients. Considerations should be taken regarding the type of diet delivery patients receive in the hospital and in rehabilitation. 

Additionally, estimated energy requirements should be calculated for each stroke patient, taking age, gender, and BMI into account, to ensure that portions are appropriate and customized. Regarding BMI, a phenomenon known as the “obesity paradox” has been demonstrated in stroke patients, by which patients who have a BMI of >30 have lower post-stroke mortality rates than non-obese patients. In the acute phase, this has been demonstrated in a Spanish nationwide database study, in which Barba et al. found that obese patients were at a lower risk for in-hospital mortality after stroke (OR = 0.71; 95% CI = 0.67–0.76) than non-obese patients [48]. In the chronic phase, a Danish nationwide study likewise demonstrated lower mortality rates at an average of 1.5 years follow-up for overweight and obese patients compared to normal-weight patients, while underweight patients had higher mortality rates [49]. The mechanism of action regarding the obesity paradox remains unclear, although hypotheses regarding the presence of adipose tissue as protective against oxidative stress have been proposed [50].

## 4. What Role does Dysphagia Play in Post-Stroke Nutritional Status?

It is well documented in the literature that dysphagia is one of the most common challenges for a post-stroke patient, with incidence rates reported between 7–65% across review articles on the subject [51,52,53]. This wide range of incidence is likely due to the difference in study designs and populations. Dysphagia carries a large risk of aspiration in the hospital and rehabilitation setting, for which methods such as videofluoroscopic swallow studies (VSS) have been proposed to monitor aspiration risk [54].

Additionally, dysphagia introduces hydration and nutritional difficulties for the stroke patient. Patients with mild to moderate dysphagia may receive a “dysphagia diet,” which consists of thickened liquids and foods with a smoothened texture [55], while severe dysphagia patients may require tube feeding. Regarding hydration, some evidence suggests that the viscosity of beverages fed to stroke patients is inversely related to the quantity of fluid that the patients consume, which is likely due to swallowing difficulties. McGrail and Kelchner found that hospitalized stroke patients who received thin liquids consumed significantly more fluid than those who received thickened liquids (*p* = 0.04), although the vast majority of both groups did not meet a minimum of 1500 mL/day [56]. Providing dysphagic stroke patients with the requisite nutrition given aspiration risk and the tendency of dysphagia patients to resist thickened liquids is challenging. In a retrospective study of 261 post-stroke patients consisting of non-dysphagic patients on a normal diet, mild dysphagic patients on a dysphagia diet, and severely dysphagic patients on tube feeding, Kim et al. found that the latter two groups were malnourished according to their prealbumin, albumin, protein, and lymphocyte levels [57].

Rehabilitation swallowing therapy (RST), which consists of a combination of imposing dietary changes, coaching the patient on head position during swallowing to prevent aspiration, oral exercises, and thermal stimulation has been used for post-stroke dysphagia [58,59,60].

Additionally, some recent data suggests that novel rehabilitation methods such as neuromuscular electrical stimulation (NMES) may also be effective in improvement of post-stroke dysphagia. In a case-series by Sun et al., 29 patients with moderate to severe dysphagia after stroke who received both RST and 2–3 weeks of NMES sessions improved significantly in the functional oral intake scale (FOIS) at 6 month and 2-year follow-up compared to baseline. However, this study lacked a control group [61]. In a RCT by Permsirivanich et al., 23 dysphagic stroke patients were assigned to either an RST or an NMES group for a four-week treatment period consisting of daily interventions with two-day breaks every five days. At the end of treatment, the NMES group had a 3.17 ± 1.27 increase in FOIS versus a 2.46 ± 1.04 increase in the RST (*p* < 0.001) [58]. While there is insufficient evidence other than the evidence provided in the Permisirivanich study to conclude that NMES alone is superior to RST, a recent meta-analysis of eight studies by Chen et al. concluded that NMES plus RST is superior to RST [62].

## 5. Is Nutritional Supplementation Associated with Better Validated Stroke Outcomes?

With the evidence suggesting that acute and subacute stroke patients often consume less than the requisite number of calories in the hospital and rehabilitation setting, many investigators have studied whether nutritional supplementation confers any benefit in this patient population. The Mini-Mental State Examination (MMSE), the NIH Stroke Scale (NIHSS), Functional Independence Measure (FIM), Barthel Index (BI), and the modified Rankin Scale (mRS) have been utilized by various studies to look at the functional and cognitive outcomes. Many studies found that an improvement in nutritional status correlated with a stronger performance on one or more of these measures.

In a double-blind randomized control trial of 48 stroke patients with subacute stroke by Aquilani et al. in 2008, an experimental group was given a daily nutritional supplement of 250 kcal + 20 g protein in addition to baseline diet and compared with a control group of baseline diet for a three-week period. At the 21-day mark post intervention, the MMSE of the experimental group was significantly improved compared with the control group (*p* = 0.01), suggesting that protein and calorie supplementation may improve cognitive recovery in subacute stroke patients [63]. In another study by Aquilani et al. in 2008 with a similar cohort of 42 patients, a protein supplemented group was found to have a larger improvement (i.e., a decrease) in NIHSS scores compared with the control group, with the protein supplemented group experiencing a decrease of 4.4 ± 1.5 versus 3 ± 1.4 in the control group (*p* < 0.01) [33]. Additionally, the study found that carbohydrate/protein intake ratio was directly related to NIHSS score while protein intake was inversely related to NIHSS score, suggesting that increased protein intake was associated with larger improvements in post-stroke patients [33].

Another study by Rabadi et al. randomized 102 undernourished subacute stroke patients to either standard feeding or intensive feeding consisting of a 120 mL supplement containing 240 calories, 11 g of proteins every 8 h. The patients in the intensive feeding group had an average Functional Independence Measure of 31.49 versus 22.94 in the standard feeding group (*p* = 0.001). Additionally, the intensive feeding patients scored significantly better in the 2-minute and 6-minute walk test, a functional test that measures the distance covered in these allotted time intervals [31].

In a single-blind randomized controlled trial by Ha et al., 170 patients with acute stroke who were deemed at nutritional risk via the Malnutrition Universal Screening Tool (MUST) were randomized to either a normal hospital diet (control group) or an individualized diet (experimental group). The individualized diet was determined by measuring energy requirements of each patient and providing protein and calorie rich diets to accommodate each patient. At a 3-month follow-up, a significantly lower proportion of the experimental group had lost ≥5% body weight (*p* = 0.05), and the experimental group also had a significantly higher quality of life score (*p* = 0.009). Of note, the trial lost 27% of the enrolled patients at follow-up.

Despite the encouraging results of the aforementioned studies regarding nutritional supplementation improving stroke outcomes, the largest RCT to date examining the impact of nutritional supplementation, the Feed Or Ordinary Diet (FOOD) Trials, found no absolute benefit to supplementation. The first of the trials, Trial 1, enrolled 4023 patients across 125 hospitals and randomly allocated patients to a control group of a normal hospital diet or an intervention group receiving the normal hospital diet plus a supplement of 360 mL of 1.5 kcal/mL, 20 g of protein per day for each day until discharge [64]. The trial found that there were no significant differences in the risk of death or poor outcome as measured by mRS 3–5 at 6-month follow-up between the two groups [64]. However, of note is that the trial did not specifically target malnourished patients.

Overall, in malnourished stroke patients, the evidence suggests that nutritional supplementation may play a beneficial role in stroke rehabilitation as measured by various outcomes measures, such as the Mini-Mental State Examination (MMSE), the NIH Stroke Scale (NIHSS), Functional Independence Measure (FIM), Barthel Index (BI), and the modified Rankin Scale (mRS). Though many studies cite NIHSS as an outcome measure when assessing nutritional supplementation, there is a limitation to its use. The scale was designed for use in clinical trials to assess modified neurological status in a simplified and reproducible manner during the acute phase [65]. Scales such as the MMSE, FIM, BI, and mRS more reliably assess the functional status of stroke patients over time and are more useful tools to evaluate nutritional supplementation on burden of disease. However, in well-nourished patients, these benefits may be absent.

## 6. Do Vitamins and Minerals Play a Role in Functional Recovery in Stroke?

Numerous studies have evaluated a variety of nutrients and mineral supplementations such as potassium, magnesium, Vitamins A, B, C, E, D, and Omega-3 Fatty Acids [66,67]. Vitamin and mineral supplementation can serve both neuroprotective and neurorecovery roles. For patients recovering from stroke, increased vitamin and mineral intake can improve antioxidant capacity and increase functional recovery [68]. Aquilani et al. discusses the supportive role of supplementary antioxidants, which include vitamin C and vitamin E, to reduce oxidative damage. Multiple studies found stroke patients to have lower levels of vitamin C and vitamin E and evaluated the effect of oral daily supplementations by randomly assigning patients to either receive or not receive supplementation. Increased dietary intake of vitamin C and vitamin E improved antioxidant capacity, and thereby provided an anti-inflammatory effect due to lower levels of C-reactive protein (CRP) and plasma malondialdehyde [68]. 

Despite the protective intent of inflammation, chronic inflammation may result in trauma and hinder the recovery process due to the presence of pro-inflammatory molecules and leukocyte recruitment [67,69]. Controlling the inflammatory response post stroke would facilitate prevention of secondary ischemic injury and enhance patient rehabilitation [70]. Anti-inflammatory dietary components can greatly influence the process of inflammation by reducing levels of CRP and other inflammatory markers, such as interleukin (IL)-1, IL-6, or tumor necrosis factor alpha (TNF-α). Consequently, a favorable reduction of pro-inflammatory markers results in endothelial protection. Adopting a diet with antioxidants, such as polyphenols and flavonoids, from fruits, berries, vegetables, nuts, and whole grains can impart beneficial anti-inflammatory effects. Other anti-inflammatory dietary factors, such as high soluble and insoluble fiber content, can complement a diet high in antioxidants [67]. Addition of herbs and spices, such as chili pepper and basil, can also induce anti-inflammatory effects by reducing IL-6 production and elevating IL-10 production [71].

In addition to vitamin C and vitamin E, other clinical evidence supports vitamin B and vitamin D to improve stroke recovery. In a randomized control trial by Pan et al., a total of 283 participants were enrolled in a randomized, double-blind, placebo-controlled trial of vitamin B supplementation. Participants assigned to vitamin B supplementation received daily folic acid (2 mg), vitamin B6 (25 mg), and vitamin B12 (0.5 mg). Post stroke survivors who received vitamin B supplementation were associated with lower hazard of major depression of 0.48 (95% CI, 0.31–0.76) compared to the placebo participants. Almeida et al. reported the positive effects of long-term treatment with vitamin B for stroke patients in reduction of major depression and improved mental well-being [72].

Vitamin D plays a neuroprotective, neuromuscular, and osteoprotective role, and low 25(OH)D levels have also been associated with cerebrovascular risk factors [73]. Alternatively, vitamin supplementation can also serve a secondary role in strengthening post stroke physical status and preventing other complications that may worsen disability and rehabilitation efficiency [68,74,75]. Elderly hemiplegic stroke patients with low serum 25(OH)D concentrations are prone to greater risk of hip fracture and may benefit from vitamin D supplementation to prevent reduction in bone mineral density [74].

Focus on mineral supplementation, such as potassium and magnesium, is another component of nutritional management. In a randomized, multicenter, double-blind controlled trial, Pan et al. assigned 291 discharged stroke patients into three groups: regular sodium (Na) salt (*n* = 99), potassium-enriched (K) salt (*n* = 97), and potassium- and magnesium-enriched (K/Mg) salt (*n* = 95). After a 6-month intervention, neurological performance was evaluated using the NIH Stroke Scale (NIHSS), Barthel Index (BI), and modified Rankin scale (mRS). Long-term delivery of potassium and magnesium to meet dietary reference intake (DRI) values was found to enhance neurological recovery in stroke patients. In comparison with the Na salt group, the K/Mg salt group displayed a significant improvement in neurologic performance. Interestingly, compared to the Na salt group, the K salt group did not display a significant improvement in neurologic performance [75].

Sahota and Savitz highlight the numerous pathways magnesium may impact, including increased blood flow to ischemic brain areas and improved recovery of cellular energy metabolism after ischemia. Numerous pilot studies and clinical trials support the neuroprotective effects of magnesium, however, to varying degrees [76]. The varying results of magnesium supplementation may reside in the different trial environments and designs. Sutherland et al. reviewed studies involving magnesium supplementation studies and found more promising results when studies were not temperature controlled and did not delay supplementation after stroke onset [77].

The wide-ranging benefits of vitamin and mineral supplementation for stroke patients appear promising to improve rehabilitation outcomes. Of these, the strongest evidence exists for vitamin C, vitamin E, potassium, magnesium, and omega-3 fatty acids. Other forms of supplementation and nutritional counseling and intervention have been associated with positive outcomes. Supplementation may promote overall physical health and mental health, both of which are important for quality of life and high rehabilitation efficiency.

## 7. Does Motivational Coaching and Self-Efficacy Impact the Likelihood of Adhering to a Stroke Diet?

Rehabilitation from a stroke can cause a tremendous burden for both the patient and members of the patient’s family. In recent years, additional emphasis has been placed on providing longitudinal coaching to chronic stroke patients. There is emerging evidence that this method of support is effective for helping patients maintain a healthy post-stroke diet. Additionally, there is evidence that a stroke survivor’s internal drive, resilience, and self-efficacy play a role in adopting good behaviors.

A randomized controlled trial by Gillham et al. tested the efficacy of “enhanced secondary prevention,” which consisted of providing patients with additional counseling, motivational interviewing, and frequent telephone follow-ups after suffering a minor stroke [78]. Of the 52 patients enrolled in the trial, half received this intervention, while the control group received “conventional care.” On average, patients in the “enhanced secondary prevention group” increased their consumption of fruits and vegetables by 7.6 servings per week, while the patients in the control group only increased 2.0 servings per week (*p* = 0.03), indicating that coaching can have a significant impact on stroke patients’ dietary choices [78]. Similarly, a study by Ovbiagele et al. offered a longitudinal coaching intervention to hospital patients who presented with acute transient ischemic attack (TIA) or ischemic stroke, which included a dietary component with “at least 5 servings of fruits and/or vegetables per day, at least 2 servings of fish per week, and at least 1 fiber-rich meal per day; less than one-third of daily intake attributable to fat.” Of the 130 patients for whom there was 90-day follow-up, adherence to the dietary component was 78% [79]. However, a drawback to this study is that there was no comparison group that did not receive intervention.

In addition to coaching interventions, there is evidence that a stroke patient’s behavioral characteristics can influence one’s long-term adherence to dietary guidelines. In a prospective cohort study of 100 patients with either transient ischemic attack or ischemic stroke, Brouwer et al. found that a baseline of self-efficacy, as determined by patient questionnaires, was the strongest predictor of a patient’s intention to adapt a healthy diet (95% CI, 0.23–0.75) [80]. Based on the aforementioned studies, it may yield benefit for hospitals’ neurosurgery and neurology departments to coordinate long term stroke coaching programs and assess patients’ behavioral patterns to increase the probability of patients adhering to healthy lifestyles. 

## 8. Conclusions

There is strong evidence that malnourishment is common in the post-stroke hospital and rehabilitation setting, with a large proportion of the patients not reaching their estimated average requirements. Malnourishment in these patients can be caused or exacerbated by dysphagia, with dysphagia therapy ranging from coaching to neuromuscular electrical stimulation (NMES). There is some disagreement around whether the resting energy expenditure (REE) is altered in acute and subacute stroke patients, with most evidence pointing to there being no meaningful change in REE, although small sample sizes and variable study designs limit applicability of these studies. Regarding the utility of nutritional supplementation in acute and subacute stroke patients, while the largest trial to date (FOOD) found no benefit to supplementation, smaller studies focused specifically on malnourished cohorts have found that protein-calorie supplementation leads to improvements in patients’ cognitive and functional recovery post-stroke. Additionally, there is modest evidence pointing to the post-stroke supplementation of vitamins and minerals for preventing the occurrence of depression and bone fractures, and allowing for increased blood flow to ischemic areas. Post-stroke coaching programs have been shown to lead to higher adherence to healthy diets and lifestyles.
